# Research on Dewatering Characteristics of Waste Slurry from Pipe Jacking Construction

**DOI:** 10.3390/ma15062242

**Published:** 2022-03-18

**Authors:** Libing Jiang, Liang Zhen, Jianfeng Wang, Tao Zhang, Xianwen Huang

**Affiliations:** 1 Water Supply and Drainage Management Office, Zhenjiang 212008, China; lbjiang1985@yahoo.com; 2 Shanghai Road and Bridge (Group) Co., Ltd., Shanghai 200433, China; wjf7907@126.com (J.W.); zhangtao554@126.com (T.Z.); 3 School of Civil Engineering and Architecture, Anhui University of Science and Technology, Huainan 232001, China

**Keywords:** waste slurry, flocculation, solidification, pipe jacking

## Abstract

A large amount of waste slurry is produced during the construction of pipe jacking projects. To avoid the waste slurry occupying too much urban land, it needs to be rapidly reduced. Due to the complex composition of waste slurry, the existing dewatering methods face the problem of low efficiency, and the soil after dewatering is difficult to recycle as soil materials due to high water content and low strength. There is currently a lack of research on dewatering and resource utilization of waste slurry from pipe jacking projects. In response to this problem, this paper studies the flocculation-settling characteristics of waste slurry and the mechanical properties of solidified sediment. It was found that the anionic polyacrylamide (APAM) 7126 obtained the best separation effect if the waste slurry contains bentonite, which increases the zeta potential, resulting in poor separation. Thus, FeCl_3_·6H_2_O and APAM 7126 can be used as compound conditioners. The sediment after settling was further added with 20–30% sulphate aluminum cement (SAC), and the unconfined compressive strength of the solidified sediment for 3 days could exceed 30 kPa. After flocculation-settling and solidification treatment, the waste pipe jacking slurry can be quickly dewatered into a soil material with a certain strength, which provides a reference for engineering applications.

## 1. Introduction

As a trenchless technology, pipe jacking is increasingly used in the construction of urban sewage and rainwater pipe networks [1,2]. Slurry is an indispensable material in pipe jacking projects, which plays the role of balancing the pressure of the excavation surface, discharging the slag, and lubricating [1,3]. During the construction process, a large amount of waste slurry is continuously produced, which is likely to contain bentonite, carboxymethyl cellulose (CMC) and other components [1,4,5]. These components may affect the separation results, thus requiring more site on which to dispose of the slurry. With the development of urbanization, there are fewer sites that can be used as slurry storage. It is necessary to progress the rapid reduction and resource utilization of waste slurry.

Flocculation was initially used in water treatment [6,7,8], and was gradually applied in the dewatering of slurry, such as waste dredging slurry [9,10] and waste tailings slurry [11,12]. Suitable flocculation pretreatment can increase the floc size in slurry and improve the separation efficiency [9]. Wang et al. [13] used various types of polyacrylamide (PAM) to treat construction waste slurry. After 7 days of settlement, the water content of the slurry can be reduced to about 80%. He et al. [14] used cationic PAM to treat waste slurry and, after 10 min of setting, the volume of slurry can be reduced by 60%. However, the properties of pipe jacking slurry are special, and it is likely to contain bentonite, CMC, and so forth. These special components may have a potential impact on the flocculation and separation of pipe jacking slurry, and corresponding research needs to be carried out. In addition, there are many types of commercial flocculants on the market—it is unclear which flocculant has the best flocculation effect and needs to be studied.

During the flocculation process of slurry, soil particles aggregate to form flocs [15], which increases the soil–water separation rate. However, due to the limitation of storage site space, the time of settlement cannot last too long. The water content of sediment remains high after a short period of settling [13,16], resulting in poor mechanical properties and cannot be used as soil materials. In some projects, plate and frame filter presses have been used to dewater the slurry, which can greatly reduce the water content of slurry [17,18]. However, the dewatering efficiency of the filter press is low in some cases, and the problem of untimely treatment often occurs [19]. Untimely reduction leads to no place to store excess waste slurry, resulting in the stagnation of construction. Solidification technology is often used to increase the strength of soft soil [20,21,22,23] and has been applied in some projects [24]. The curing agent converts the excess free water into mineral water through hydration, which improves the mechanical properties of soft soil. It may be possible to further treat the flocculated sediment by means of solidification, which could increase the strength of sediment to make it a reusable soil material. The solidification characteristics of sediment obtained by slurry flocculation and settling are still lacking, and need to be studied further.

Based on the problem of unclear flocculation, the sedimentation characteristics of waste pipe jacking slurry and the unclear strength growth law of the solidification sediment after sedimentation, this paper carried out relevant laboratory experiments. The waste slurry produced in two different stages of pipe jacking construction was selected as the experimental material. Five different conditioners were used to pretreat pipe jacking slurry, and the sedimentation characteristics were studied. For the sediment obtained by settling, two solidification agents were used to conduct solidification experiments to study mechanical properties of the solidified sediment. Based on the above tests, the flocculation-settling characteristics of pipe jacking waste slurry were studied, and the feasibility of using a flocculation–sedimentation and solidification combined method to treat waste pipe jacking slurry was discussed, to provide a reference for related projects.

## 2. Materials and Methods

### 2.1. Slurry

**Figure 1:**
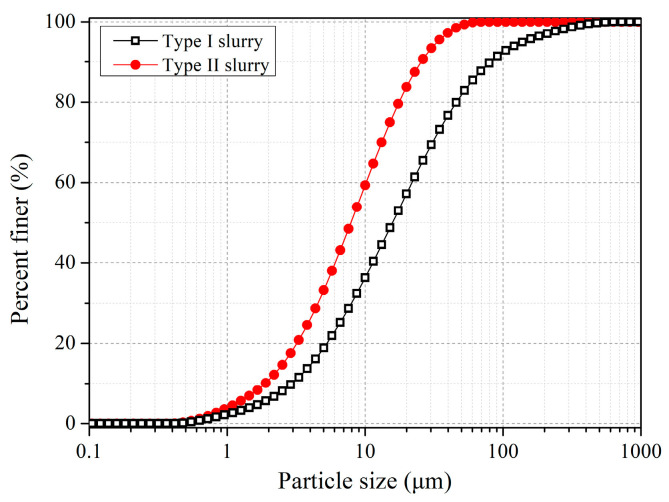
Particle size distribution of the pipe jacking waste slurry.

In order to study the flocculation–sedimentation and solidification characteristics of waste pipe jacking slurry, waste slurry from a pipe jacking project in Zhenjiang, Jiangsu Province, China, was used as the test material. The sampling of the waste slurry at the site was carried out twice. The first sampling time was on 4 January 2021 (Type I slurry), and the second was on 26 March 2021 (Type II slurry). According to the construction report of the pipe jacking project, the construction was excavated to a special stratum in March 2021. To maintain the stability of the excavation surface, more bentonite was added to the slurry. Therefore, Type II waste slurry was mixed with some bentonite. Table 1 shows the basic properties of the pipe jacking waste slurry. Particle size distribution of the slurry was measured by a laser particle size analyzer, the Malvern Mastersizer 2000. The slurry was diluted to a water content of 300%. The sample was dropped into the Hydro MU dispersion unit through an eyedropper, and was dispersed by ultrasound and stirring. The obscuration parameter was kept between 10% and 20% to ensure good quality of the signal. The pump speed was set as 2000 r/min. The particle size distribution of waste pipe jacking slurry can be seen in Figure 1. Type II slurry has a smaller particle size because it contains bentonite, with an average particle size of 7.84 µm, while the average particle size of Type I slurry is 15.78 µm.

### 2.2. Conditioners

Four types of commercial polyacrylamide (PAM) were used for flocculation, manufactured by Shanghai Wshine Chemical Co., Ltd., Shanghai, China. Four types of PAM were recommended by the manufacturer because of their good effect in the reduction of tailings slurry. Wshinefloc 412VS and 611HN are cationic polyacrylamide (CPAM). Wshinefloc 7126 and 720VJ are anionic polyacrylamide (APAM). The specific parameters of PAM are shown in Table 2. The flocculant was reconstituted every day, at a concentration of 0.1% (*w*/*w*).

Ferric chloride (FeCl_3_·6H_2_O) was used as a coagulant in composite conditioning, which is produced by Shanghai Yuanye Biological Co., Ltd., Shanghai, China, and its molecular weight is 270.3 g/mol.

### 2.3. Solidification Agent

The 425# ordinary Portland cement (OPC) and sulphate aluminum cement (SAC) were selected as the solidification agent. SAC has a faster hydration rate than OPC, which can make the solidified soil achieve a higher strength at a shorter curing age. SAC was used to test whether it can make the sediment reach the required strength earlier in solidification experiments.

### 2.4. Flocculation-Settling Experiment

The water content of slurry was initially adjusted to 300% by adding tap water, which was used to simulate the initial state of slurry produced by pipe jacking construction. Afterwards, flocculation tests were carried out. The flocculation scheme is shown in Table 3. The flocculation test was carried out in a 500 mL beaker. After adding flocculant, the slurry was stirred at 450 rpm for 2 min. The slurry after flocculation was then subjected to a sedimentation test. The clear sediment–water interface was used to evaluate the result of separation during settling, which was recorded by the scale value on the side wall of the beaker. The sediment below the sediment–water interface is considered to be the product after slurry separation. The sedimentation test lasted for 600 s, representing a short period of concentration. As a comparative test, the sedimentation test of the slurry without adding flocculant was also carried out.

During the pre-experiment, it was found that the flocculation-settling effect of Type II slurry was very poor. Thus, two types of composite conditioning experiments were designed, as shown in Table 3. Type II slurry was first added with FeCl_3_·6H_2_O and was stirred at 450 rpm for 2 min, and then added with PAM and stirred at 450 rpm for 2 min to test sedimentation results.

### 2.5. Solidification Experiment

After the flocculation-settling experiment, the sediment with the lowest water content after settling was subjected to the solidification experiment. The supernatant of the experimental group was poured out, and a solidification agent was added to the sediment for stirring, wherein the stirring speed was 450 rpm, and the stirring was performed for 2 min. Subsequently, the sediment was layered into the mold. The inner diameter and height of the mold are 39.1 mm and 80.0 mm, respectively.

According to results with PAM pretreatment in Section 3, the sediment pretreated with APAM 7126 (0.25%) had the lowest water content. For the composite pretreatment experiments, the combined addition of FeCl_3_·6H_2_O (3%) and APAM 7126 (0.10%) obtained the sediment with lower water content. Therefore, the sediment obtained from these two sets of experiments was used for solidification experiments. The solidification scheme is shown in Table 4.

The moist room YH-60B type (produced by Beijing central North Road Instrument Equipment Co., Ltd., Beijing, China) was used in the solidification experiment. The curing conditions were a temperature of 20 °C and humidity of >95%. After a set curing time, an unconfined compressive strength experiment was performed.

### 2.6. Unconfined Compressive Strength Experiment

After the designated curing time was reached, specimens were tested for the unconfined compressive strength (*q*_u_). A strain-controlled apparatus (YYW-2 type, Nanjing Soil Instrument Factory CO., LTD., Jiangsu, China) was used to test unconfined compressive strength. Each test was performed thrice in parallel.

### 2.7. Zeta Potential Experiment

After the settling experiment, the supernatant was taken out to test the zeta potential of the slurry after flocculation pretreatment. Zeta potential is often used to characterize the electrical characteristics of particles and can be used to reveal the degree to which particles are affected by conditioners. The zeta potential was measured through electrophoretic light scattering by using a Zetasizer Nano ZSP (Malvern Instruments, Malvern, UK). DTS1070 cell was used to perform the zeta potential test. The supernatant was injected into the cell, then the cell was pressed into the measurement chamber to test the zeta potential. Each test was performed thrice in parallel.

## 3. Results

### 3.1. Sedimentation Results without Flocculation

**Figure 2:**
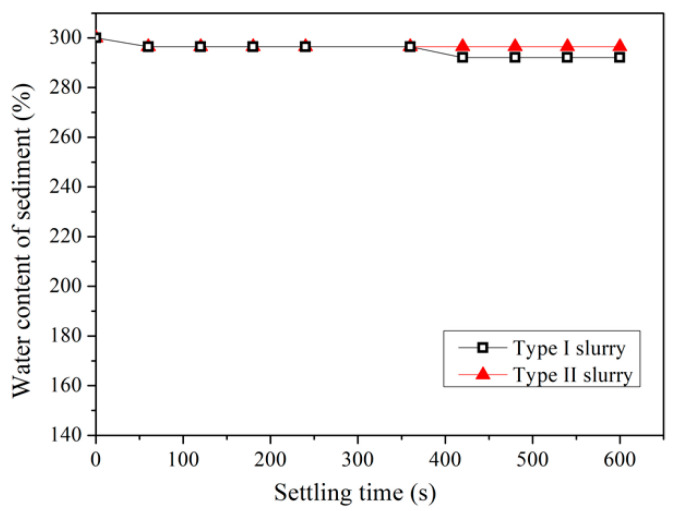
Sedimentation results of pipe jacking waste slurry without flocculation.

The pipe jacking waste slurry with an initial water content of 300% was subjected to a self-weight sedimentation test. Figure 2 demonstrates that the sedimentation and separation of two types of slurry without pretreatment are very slow, and the water content of slurry drops from 300% to 292% and 296% respectively within the settling time of 600 s. It can be inferred that if the pipe jacking waste slurry is not treated, it could occupy a large area of the site for storage. Most pipe jacking projects are in the underground areas of a city. It is obviously difficult to find a large storage space in the city to store the slurry. Therefore, it is meaningful to quickly reduce the amount of slurry.

### 3.2. Sedimentation Results with PAM Pretreatment

**Figure 3:**
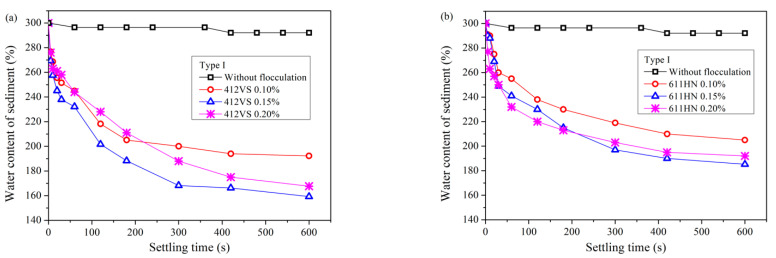
Sedimentation results of slurry (Type I) after pretreatment with CPAM. (**a**) 412VS. (**b**) 611HN.

**Figure 4:**
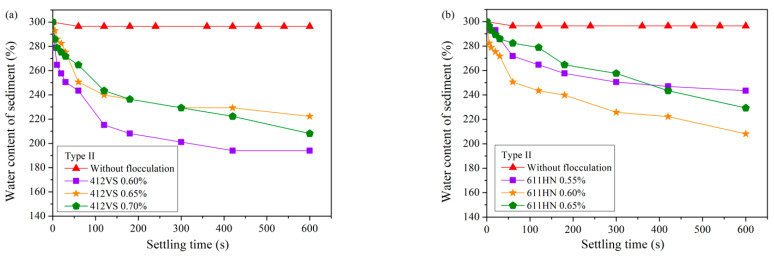
Sedimentation results of slurry (Type II) after pretreatment with CPAM. (**a**) 412VS. (**b**) 611HN.

Figure 3 and Figure 4 show the sedimentation results of the waste pipe jacking slurry after adding CPAM. Compared with the slurry without pretreatment, the addition of two types of CPAM can make the waste slurry settle and separate rapidly.

Figure 3 shows the settling results of Type I slurry. The optimal dosage of CPAM 412VS was 0.15%, and the water content of the sediment dropped rapidly from 300% to 159% within the settling time of 600 s. The CPAM 611HN also obtained good treatment results. The optimum dosage was 0.15%, and the water content of the sediment finally dropped to 185%.

Figure 4 reveals the settling results of the Type II pipe jacking waste slurry. Compared with Type I slurry, the sedimentation effect of Type II slurry containing bentonite was worse, and a higher dosage of CPAM was required for pretreatment. Using the same type of CPAM, the optimal addition amount was increased by three times, and the optimal dosage of 412VS and 611HN were both increased to 0.60%. In addition, in the same settling time, the water content of the sediment was higher, and the water content of the sediment after pretreatment with the two types of flocculants was 194% and 208%, respectively.

Comparing two types of CPAM, 412VS has a better pretreatment effect than 611HN, and the water content of the sediment can be reduced to a lower level under the same addition amount.

**Figure 5:**
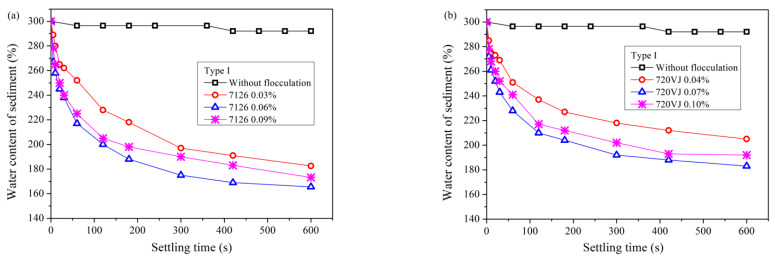
Sedimentation results of slurry (Type I) after pretreatment with APAM. (**a**) 7126. (**b**) 720VJ.

**Figure 6:**
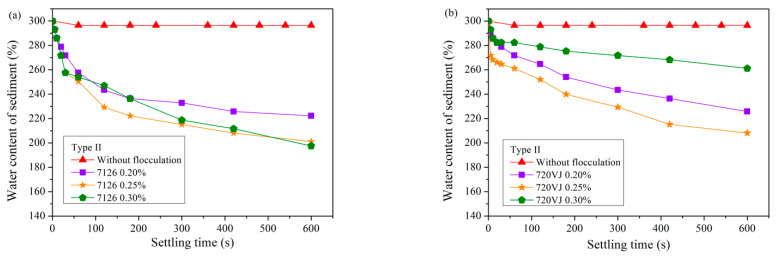
Sedimentation results of slurry (Type II) after pretreatment with APAM. (**a**) 7126. (**b**) 720VJ.

Figure 5 and Figure 6 show the sedimentation results after pretreatment of waste pipe jacking slurry using APAM. The addition of APAM also increased the speed of soil–water separation. Unlike CPAM, the optimal dosage of APAM was much lower than that of CPAM, no matter for Type I slurry or Type II slurry.

Figure 5 reveals the sedimentation results of Type I slurry. The optimal addition amount of APAM 7126 was 0.06%. After 600 s of sedimentation, the water content of sediment finally dropped to 166%. The optimum dosage of APAM 720VJ was 0.07%, and the final water content of the sediment was 183%.

Figure 6 shows the settling results of bentonite-rich slurry (Type II slurry). Similar to the results of CPAM pretreatment, the treatment of Type II slurry with APAM requires a larger amount of flocculant than Type I slurry. The optimum addition amount of APAM 7126 reached 0.25%, and the final water content of the sediment dropped to 201%. The optimum dosage of APAM 720VJ reached 0.25%, and the water content of the sediment was 208%.

### 3.3. Sedimentation Results with Compound Pretreatment

**Figure 7:**
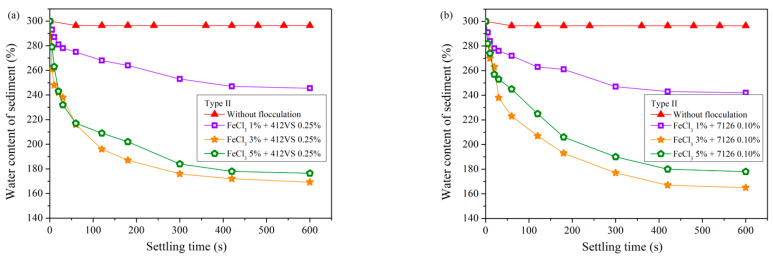
Sedimentation results of slurry (Type II) after compound pretreatment. (**a**) FeCl_3_ + 412VS. (**b**) FeCl_3_ + 7126.

Figure 7 reveals the sedimentation results of Type II slurry after compound pretreatment. Comparing Figure 7a with Figure 4a, after the compound treatment of FeCl_3_·6H_2_O and CPAM 412VS, the settling and separation effect became better. After adding 3% FeCl_3_·6H_2_O, the optimal addition of CPAM 412VS decreased from 0.60% to 0.25%. The water content of the sediment can be reduced to 169% in 600 s, which is lower than the result of the single addition of CPAM 412VS.

Similarly, Type II slurry pretreated with FeCl_3_·6H_2_O and APAM 7126 also obtained better separation results, and the optimal combination was 3% FeCl_3_·6H_2_O and 0.10% APAM 7126. Comparing Figure 7b with Figure 6b, after the compound pretreatment, the water content of the sediment can be reduced to 165% in 600 s, and the added amount of the APAM 7126 used was also reduced from 0.25% to 0.10%.

### 3.4. Solidification Results

**Figure 8:**
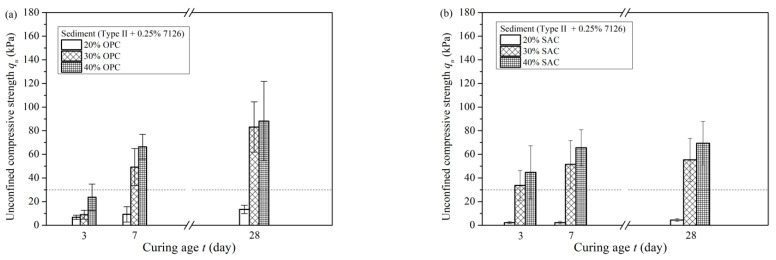
Unconfined compressive strength of the solidified sediment after APAM pretreatment. (**a**) OPC. (**b**) SAC.

In experiments where the slurry was only pretreated with PAM, the slurry treated with APAM 7126 achieved the best settling effect. The sediment obtained by settling had a lower water content, so the sediment was used for the solidification experiment. The cured samples were tested for unconfined compressive strength and the results are shown in Figure 8. Figure 8a shows the unconfined compressive strength of cured sediments with different dosages of OPC at different curing ages. With the increase of curing age, the unconfined compressive strength increased continuously. When the OPC dosage was 30%, the strength was significantly improved, the 7-day strength was 49.23 kPa, and the 28-day strength was 83.09 kPa. In the initial stage, the strength of the 3-day was only 8.95 kPa.

Figure 8b shows the unconfined compressive strength of sediments added with SAC at various curing ages. Compared with OPC, SAC could improve the strength of the cured sediment in less curing time. For the solidified sediment with 30% SAC, the 3-day strength was 33.70 kPa, and the strength nearly reached the peak value of 51.46 kPa after curing for 7 days, and the strength was 55.30 kPa at the age of 28 days.

**Figure 9:**
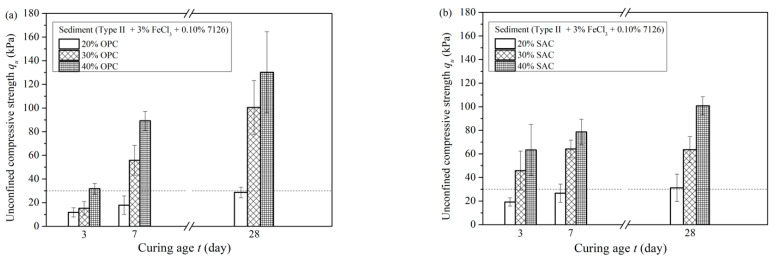
Unconfined compressive strength of the solidified sediment after compound pretreatment. (**a**) OPC. (**b**) SAC.

The slurry had better sedimentation results after the compound pretreatment of FeCl_3_·6H_2_O and APAM 7126, and the sediments were also subjected to solidification experiments. Figure 9 shows the unconfined compressive strength of the sediments. Compared with the samples obtained after the pretreatment with APAM 7126, the samples with the compound pretreatment have higher strength under the same amount of curing agent. For example, when OPC was used as the curing agent, the unconfined compressive strength at 30% was 15.2 kPa at 3 days, 55.8 kPa at 7 days, and 100.44 kPa at 28 days. In contrast, SAC can make the sample have a certain strength earlier under the same addition amount, as shown in Figure 9b. When the dosage of SAC was 30%, the strength reached 45.80 kPa, 64.2 kPa and 64.1 kPa at 3 days, 7 days, and 28 days, respectively.

In summary, the sediment separated by sedimentation has a certain strength after solidification. Both types of curing agents can improve the strength of sediment and modify the sediment into a soil material with a certain strength. The use of SAC as a curing agent can rapidly improve the mechanical properties of solidified sediment in a short period of time. The unconfined compressive strength of cured sediment is greater than 30 kPa in 3 days.

### 3.5. Zeta Potential after Pretreatment

**Figure 10:**
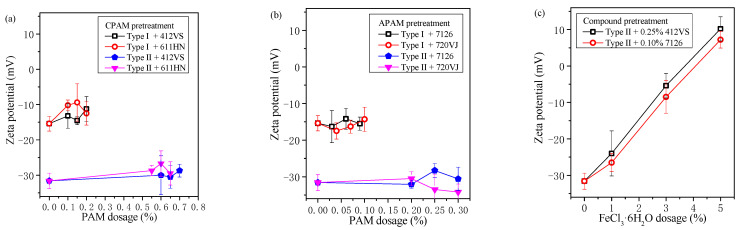
Zeta potential after pretreatment. (**a**) CPAM. (**b**) APAM. (**c**) FeCl_3_ + PAM.

The supernatants of all sedimentation experiments were used to test the zeta potential, and the test results are shown in Figure 10. The initial zeta potentials of Type I slurry and Type II slurry were quite different, which were −15.4 mV and −31.6 mV, respectively. Figure 10a,b show the zeta potentials of the slurry pretreated with CPAM and APAM, respectively. After adding CPAM or APAM, the zeta potential of the slurry did not change significantly, which indicated that the four types of polymer flocculants used in the experiment did not basically change the electrical properties of the particles in the slurry. Especially when APAM was added, the zeta potential of the slurry was basically unchanged. Combining with Table 2, this could be because APAM has a relatively low charge density, and the electrical properties of the particles in the slurry basically do not change.

Figure 10c shows the results of the zeta potential of the waste pipe jacking slurry treated with FeCl_3_·6H_2_O and PAM. With the addition of FeCl_3_·6H_2_O, the zeta potential of the slurry changed significantly. The zeta potential gradually approached 0 from −31.6 mV, and when the addition of FeCl_3_·6H_2_O reached 5%, the zeta potential changed to about 10 mV.

## 4. Discussion

### 4.1. Flocculation-Settling Characteristics of Pipe Jacking Waste Slurry

If the waste slurry from pipe jacking is not pretreated, it can be seen from Figure 2 that the sedimentation effect is very poor, and it is difficult to separate soil and water. When PAM is used as a pretreatment agent, all four flocculants can significantly improve the efficiency of slurry self-weight separation from Figure 3, Figure 4, Figure 5 and Figure 6. The water content of slurry can be rapidly reduced from 300% to 159–208%. For Type I slurry, the optimal flocculant is APAM 7126, and the optimal addition is 0.06%. For Type II slurry, APAM 7126 is also the optimal flocculant, but the optimal addition is 0.25%. The properties of waste slurry produced in different periods of the same pipe jacking project are different, resulting in great differences in the optimal dosage of flocculant and sedimentation results.

From the test results of zeta potential in Section 3.5, the zeta potential of Type II slurry after PAM pretreatment is about −30 mV, and the corresponding optimal dosage of PAM is high. The zeta potential of Type I slurry after PAM pretreatment is about −15 mV, and the optimal additional amount of PAM is low.

**Figure 11:**
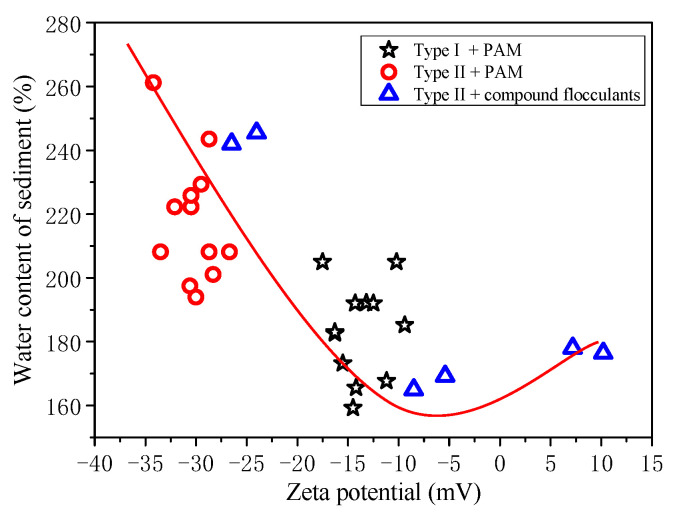
Relationship between water content of sediment and zeta potential.

The zeta potential of the slurry changes dramatically when the slurry is pretreated with compound conditioners (Figure 10c), and the optimal additional amount of PAM decreases. It can be inferred that the zeta potential of the waste slurry from pipe jacking is closely related to the sedimentation and flocculation results of the slurry. Therefore, the above experiments pretreated with PAM are analyzed, and the relationship between the water content of sediment and zeta potential after sedimentation is discussed, as shown in Figure 11.

Figure 11 clearly reveals that the water content of sediment is significantly correlated with zeta potential. After flocculant pretreatment, the closer the zeta potential is to 0, the lower the water content of the sediment is. The initial zeta potential of Type II slurry is high. After PAM pretreatment, the zeta potential is still about −30 mV, so the water content of the sediment is high. The initial zeta potential of Type I slurry is relatively low. After PAM pretreatment, the water content of sediment obtained by sedimentation is relatively low. Therefore, it is speculated that Type II slurry contains more bentonite, which increases the zeta potential of the slurry, making it difficult to flocculate and separate. In other studies [9,25], slurries rich in montmorillonite were also found to be less effective at flocculation, explained by lower zeta potential, which confirmed the findings of this paper. According to these test results, it can be inferred that the clay minerals contained in the waste slurry produced by the construction of different formations is different, and it is necessary to further study the influence of clay minerals on flocculation in detail in the future.

It is worth noting that, for Type II slurry, after pretreatment with compound conditioners, the zeta potential of the slurry changes rapidly and the water content of the corresponding sediment decreases rapidly.

Based on the above discussion, the flocculation mechanism of different PAM can be further discussed. All four PAM shown in Figure 3, Figure 4, Figure 5 and Figure 6 can accelerate the settlement of slurry. However, the addition of PAM has a very small change in the zeta potential of the slurry in Figure 10. Therefore, it can be speculated that electrical neutralization is not the main mechanism of the four PAM. The initial zeta potential of the slurry is negative, but both APAM can make slurry flocculate and settle rapidly, indicating that bridging plays a major role in flocculation. APAM 7126 achieves a better sedimentation effect than APAM 720VJ; the higher molecular weight of 7126 could be the main reason, which confirms that bridging is the main flocculation mechanism. Two CPAM have a certain charge density, and compared with the two APAMs, they have a better performance in adjusting the zeta potential as shown in Figure 10. However, the optimal amount of flocculant added is much higher than that of APAM, and its lower molecular weight may be the main reason. These results confirm that bridging is the main mechanism for PAM to flocculate particles.

The zeta potential of the slurry decreases rapidly after the addition of FeCl_3_·6H_2_O, which indicates that the FeCl_3_·6H_2_O significantly increases the strength of the ions in the slurry, thereby reducing the electrostatic repulsion between soil particles. After the subsequent addition of PAM, the PAM can be better interacted with soil particles in the slurry, thereby improving the effect of flocculation and separation. Therefore, under the action of compound conditioning, the electric neutralization and bridging mechanism work together, which greatly reduces the amount of PAM added. It can be seen from Figure 10c that when excess FeCl_3_·6H_2_O is added, the zeta potential begins to increase again, and the electrostatic repulsion between particles increases, and the effect of flocculation begins to decrease again.

In summary, in the case of slurry with difficulty in separation, compound conditioning can be used for pretreatment to reduce zeta potential and improve separation efficiency.

### 4.2. New Method for Rapid Dewatering

**Figure 12:**
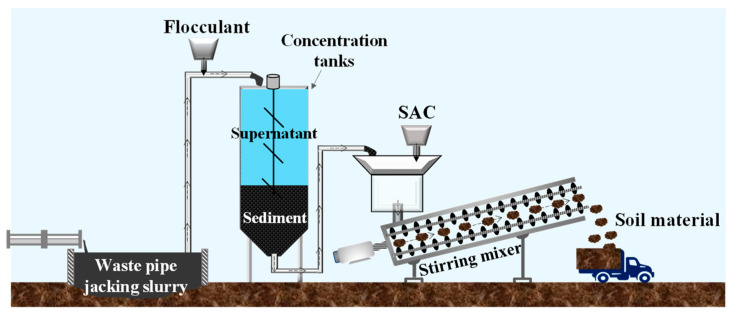
Schematic diagram of flocculation-sedimentation and solidification combined method.

Aiming at the problem that a large amount of waste pipe jacking slurry with high water content needs to be treated and utilized, a new method based on the combination of flocculation–sedimentation and solidification is proposed; the schematic diagram is shown in Figure 12.

According to the amount of bentonite added to the slurry during the construction of pipe jacking projects, FeCl_3_·6H_2_O is added appropriately to adjust the zeta potential of slurry. Then, the waste slurry is pretreated with APAM (7126), and after a short period of sedimentation (600 s or more), the water content of the sediment would be less than 165%. It is estimated that, after 600 s of sedimentation and separation, the volume of slurry is reduced to 60% of the original, which greatly reduces the occupation of the storage site by the waste slurry. The separated sediment is cured by adding a solidification agent. According to Section 3.4, adding 20–30% SAC can achieve an unconfined compressive strength of more than 30 kPa within 3 days, which meets the requirements of walking and vehicle transportation. The cured sediment can be transported to other areas for disposal and utilization.

The cost of flocculant and solidification agent by this method can be estimated. The cost depends on the nature of the waste slurry. If more bentonite remains in the waste slurry, the dewatering and reduction of the slurry will become difficult, and the addition of flocculant and curing agent will increase, resulting in the increase of the cost. Considering the most unfavorable conditions, such as Type II slurry, FeCl_3_·6H_2_O and APAM 7126 are selected as conditioners and SAC as a solidification agent. The price of conditioners and solidification agent per cubic slurry is 1.19 € and 3.02 € respectively, and the total is 4.21 €. It can be seen that the cost of adding SAC is slightly higher, which is mainly because the water content of the sediment after sedimentation is still high, which leads to the need to add more SAC to ensure the strength of solidified sediment. Therefore, if the slurry storage site in the project allows, the sedimentation time could be extended, thereby further reducing the water content of the sediment, which can reduce the amount of SAC added and reduce the construction cost.

It is worth noting that the addition of FeCl_3_·6H_2_O results in the presence of some chloride ions in the solidified sediment, and chloride ion erosion can lead to the deterioration of cement-based materials and reduce the strength of the soil [26]. The effect of chloride ions on solidified sediments, as well as finding other agents to replace ferric chloride, will be the subject of future research.

## 5. Conclusions

(1) A method for the rapid reduction and reuse of waste slurry from pipe jacking projects has been studied and proposed. Through the combined treatment of flocculation–sedimentation and solidification, the water content of the waste slurry can be reduced to about 159% in a very short time (10 min), and it can be quickly transformed into a soil material with a strength greater than 30 kPa.

(2) The properties of waste slurry from pipe jacking change with the change of excavated stratum, which affects the results of flocculation and sedimentation. The zeta potential of waste slurry containing bentonite is high, which leads to the poor result of flocculation. Through the compound pretreatment of FeCl_3_·6H_2_O and APAM, the zeta potential of slurry can be reduced, and better dewatering results can be obtained.

(3) The conditioners and solidification agents suitable for the treatment of waste pipe jacking slurry were proposed. It was found that the optimal flocculation effect was achieved when APAM 7126 (0.06–0.10%) was added. If the waste slurry contains bentonite, 3% FeCl_3_·6H_2_O can be added in advance. For sediment after flocculation and sedimentation, adding 20–30% SAC can greatly improve the strength of solidified sediment in a short period of time, which is convenient for subsequent treatment and utilization.

## Figures and Tables

**Table 1:** Basic properties of the pipe jacking slurry.

Pipe Jacking Slurry	Specific Gravity	Liquid Limit (%)	Plastic Limit (%)	Organic Matter Content (%)
Type I	2.58	42.16	22.18	4.68
Type II	2.61	71.22	32.76	4.12

**Table 2:** Chemical properties of PAM used in this study.

Flocculants	PAM Characteristics	Molecular Weight	Solid Content (%)	Charge Density (meq/g)
412VS	cationic	≥10 million	≥89	1.8–2.2
611HN	cationic	≥10 million	≥91	2.5
7126	anionic	16 million	≥89	/
720VJ	anionic	12 million	≥89	/

**Table 3:** Conditioning scheme of waste slurry.

Pipe Jacking Slurry	Water Content (%)	Conditioners	PAM Characteristics	Dosage ^1^ (%)
Type I	300	412VS	cationic	0.10, 0.15, 0.20
Type I	300	611HN	cationic	0.10, 0.15, 0.20
Type I	300	7126	anionic	0.03, 0.06, 0.09
Type I	300	720VJ	anionic	0.04, 0.07, 0.10
Type II	300	412VS	cationic	0.60, 0.65, 0.70
Type II	300	611HN	cationic	0.55, 0.60, 0.65
Type II	300	7126	anionic	0.20, 0.25, 0.30
Type II	300	720VJ	anionic	0.20, 0.25, 0.30
Type II	300	FeCl_3_ + 0.25% 412VS	cationic	1, 3, 5 ^2^
Type II	300	FeCl_3_ + 0.10% 7126	anionic	1, 3, 5

^1^ The dosage is calculated as the ratio of the dry mass of conditioner to the dry mass of slurry. The dosage is determined by pre-experiment. ^2^ 1%, 3%, and 5% are the dosage of FeCl_3_·6H_2_O, which are determined by pre-experiment.

**Table 4:** Solidification scheme of sediment.

Conditioners	Solidification Agent	Dosage ^1^ (%)	Curing Time (day)
0.25% 7126	OPC	20, 30, 40	3, 7, 28
0.25% 7126	SAC	20, 30, 40	3, 7, 28
3% FeCl_3_ + 0.10% 7126	OPC	20, 30, 40	3, 7, 28
3% FeCl_3_ + 0.10% 7126	SAC	20, 30, 40	3, 7, 28

^1^ The dosage is calculated as the ratio of the dry mass of solidification agent to the dry mass of slurry.

## Data Availability

The data presented in this study are available on request from the corresponding author. The data are not publicly available due to data which also forms part of an ongoing study.
